# Correction: Osterwalder et al. Acute Abdominal Pain: Missed Diagnoses, Extra-Abdominal Conditions, and Outcomes. *J. Clin. Med.* 2020, *9*, 899

**DOI:** 10.3390/jcm12041403

**Published:** 2023-02-10

**Authors:** Isabelle Osterwalder, Merve Özkan, Alexandra Malinovska, Christian H. Nickel, Roland Bingisser

**Affiliations:** Department of Emergency Medicine, University Hospital Basel, 4031 Basel, Switzerland

## Error in Post Code

In the original publication [1], there was a mistake in Affiliation as published. The corrected affiliation appears below:

Department of Emergency Medicine, University Hospital Basel, 4031 Basel, Switzerland

## Text Correction

There was an error in the original publication regarding the calculation of extra-abdominal causes for abdominal pain. The prevalence of extra-abdominal causes should be calculated as follows: in 162 of 480 (34%) patients, an extra-abdominal cause was the main reason for the presentation of abdominal pain. However, the prevalence of extra-abdominal diagnoses overall was higher: 239 of 556 (43%) diagnoses were of extra-abdominal origin.

A correction has been made to **Results**, *3.4. Extra-Abdominal Diagnoses*:

In total, 239 (43%) extra-abdominal diagnoses were made, the most frequent ones being of urogenital and pulmonary origin. Nineteen (5%) AP presentations were due to pulmonary problems in younger patients, with 20 (11.2%) of these being in patients 65 years old and older. Psychiatric diagnoses (F-codes according to ICD-10) were made in 17 (4.5%) cases in younger patients, compared to 3 (1.7%) cases in older patients. Three different pulmonary entities were among the top 20 underlying conditions. Six (1.6%) AP presentations were due to underlying cancer in younger patients, compared to nine (5%) in older patients. Of all 15 presentations due to cancer, 4 were due to a newly diagnosed cancer, and 11 were due to the relapse, deterioration, or complications of a previously known cancer. Twenty diagnoses were responsible for 63% of all diagnoses (see Table 2).

The authors apologize for any inconvenience caused and state that the scientific conclusions are unaffected. This correction was approved by the Academic Editor. The original publication has also been updated.
